# Replicating carbonaceous vug in synthetic porous media

**DOI:** 10.1016/j.mex.2018.07.018

**Published:** 2018-07-29

**Authors:** Hasan J. Khan, David DiCarlo, Maša Prodanović

**Affiliations:** The University of Texas at Austin, Austin, TX 78712, United States

**Keywords:** Vug replication in synthetic core, Micro-CT, 3D–printing, Synthetic glass bead media, Vug

## Abstract

This paper presents an alternative method of creating vuggy glass-bead core proxies, which can be used to investigate the effects of pore-scale features on carbonate petrophysical properties. Carbonates are complex rocks having a widespread variation in pore type, size, distribution, and porosity. With this method we can control vug shape, size, and position.

Homogeneous glass bead core proxies are sintered using 1.0 mm diameter glass beads in a muffle furnace. Vugs are 3D–printed in plastic and used to make a mold in Play-Doh^®^; which is cast in gypsum cement and used as a placeholder during the sintering process. The gypsum vug dissolves during acid flood, leaving an empty space inside the glass matrix. Computed tomography (CT) scans are made of the acid washed vug space and compared to the 3D model.

## Highlights

• Alternative method for creating vuggy glass-bead core proxies
• Higher degree of control and precision in shaping the vug, and making it similar to what is seen in nature.
• The process is repeatable.

## Graphical abstract

**Figure:**
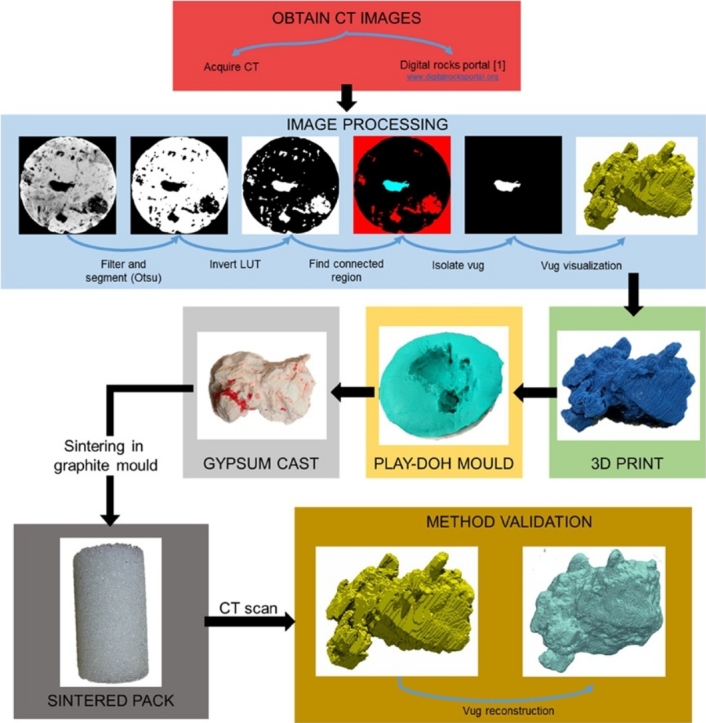

##  

**Specifications Table**

**Table:** 

Subject area	• *Earth and Planetary Sciences* • *Engineering*
More specific subject area	*Synthetic core fabrication*
Method name	*Vug replication in synthetic core*
Name and reference of original method	*Khan* et al. *(2018) [in review]*
Resource availability	

## Introduction

### Materials

• Carbonate (vug) images
• Fiji-ImageJ software
• 3D–printer
• Plaster of Paris
• Play-Doh^®^
• 1.0 mm soda lime glass bead
• Graphite mold
• Muffle furnace
• 2 M Hydrochloric acid

### Critical points

- Vug is isolated from image stack
- Vug is 3D–printed at the desired resolution
- 3D–print is used to create mold in Play-Doh^®^ which is casted with Plaster of Paris
- Vug cast is placed in bead pack before sintering
- Cooled sample is acid treated for 10 hours

## Method details

### Image acquisition

Vuggy carbonate is imaged using a medical CT scanner at a resolution of 250 μm. The scanning parameters used are: scanning time 3 s, scan thickness 1 mm, voltage 140 kV and current 200 mA. Alternatively, CT image stack can be obtained from online rock repositories [1].

### Image processing to isolate vug

CT output data is used to generate image files (e.g. JPEG etc.). The image files are imported in Fiji-ImageJ [2] where they are scaled to incorporate the difference in resolution in the X, Y and Z-axis. The image stack is filtered using the kuwahara filter to reduce noise and amplify the vug space. The stack is segmented (simple threshold command in ImageJ) using the Otsu method [3] and the look-up table (LUT) for the resultant binary stack is set such that the vug space has maximum value (255 for 8-bit image).

### Vug surface extraction

ImageJ’s built-in find connected region algorithm is then used to find the connected spaces in 3D. The algorithm color codes and isolates the individual connected spaces. The desired vug space is selected and visualized in the built-in 3D viewer, and the surface (STL) is exported for 3D–printing.

### 3D–Printing

A 3D–printer, with a minimum resolution of 100 μm, is used to print the surface. This is feasible for our print job which has a length of 30 mm, but can be a limiting factor for smaller samples. High resolution 3D–printers, with a resolution of up to 20 μm, can be used instead.

### Mold creation

The printed sample is coated in grease and pressed between two sheets of Play-Doh^®^. The Play-Doh^®^ surface is coated with grease before pressing and are pulled apart afterwards. The sample is pulled from the Play-Doh leaving a mold behind. The Play-Doh^®^ releases easily because of the applied grease. Two holes are drilled in the mold which connect the inner body to the outer surface. These can be simply made poking a pencil through the Play-Doh^®^. They are created to give a pathway to the air which escapes when fluid is poured inside the mold. Grease is applied to the template on the two sheets and they are joined together.

The mold can also be 3D–printed but we were not successful with making it leak proof.

### Die casting

Plaster of Paris (gypsum cement) is mixed with water and cast inside the mold. The cement solidifies in 30 minutes, after which the Play-Doh^®^ is removed. Excess Play-Doh^®^ is removed by blowing with air.

### Core fabrication

Glass bead core is fabricated using the method outlined in [4]. 1.0 mm glass beads are poured in a graphite mold and the vug cast is placed at the desired depth. The mold is exposed to a peak temperature of 725 °C a muffle furnace. The peak temperature is chosen to be higher than the softening point of soda-lime glass, at which the glass beads begin to soften up and join together. The system is allowed to cool before the sintered pack is removed from the mold.

### Acid treatment

The core is flooded for 10 hours with 2 M HCl, which dissolves the gypsum cement. 10 pore volumes of de-ionized water is then cycled to clean up any solid remnants. The core is placed in a drying furnace (∼150 °C) for 2 hours to remove any fluid.

## Method validation

**Fig. 1:**
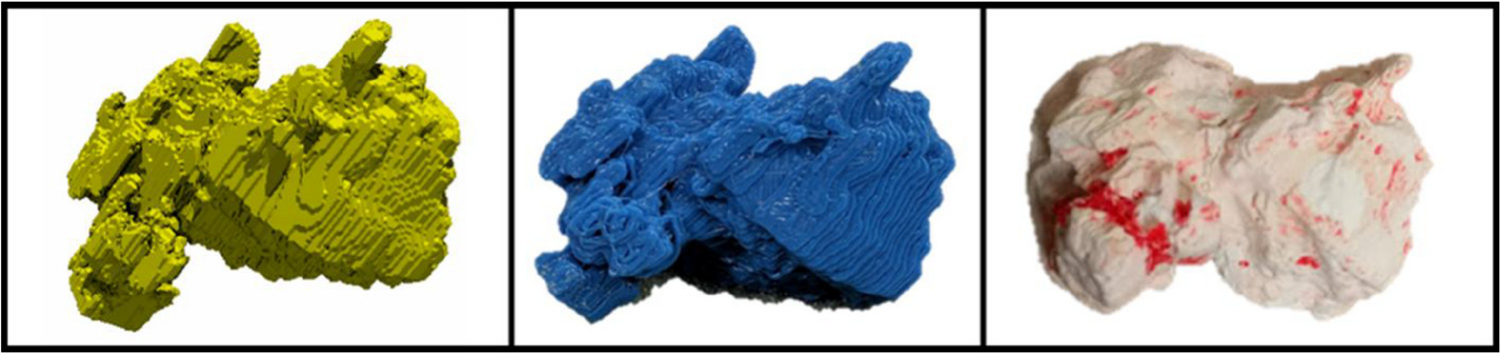
Surface visualization (left), resultant 3D–print (middle) and vug cast (right).

A Guelph dolomite sample (38 mm × 72 mm) is scanned at a resolution of 250 μm [5] and thickness of 3 mm in a medical CT scanner (Universal Systems HD-350E located at UT PGE). The images are processed to isolate a vug and surface extracted. MakerBot^®^ Replicator 2 (located at UT PGE) is used to 3D–print the surface. Mold is created and cast in gypsum cement (Fig. 1). Core is sintered with the casted vug in place and treated with acid to remove the gypsum cement.

**Fig. 2:**
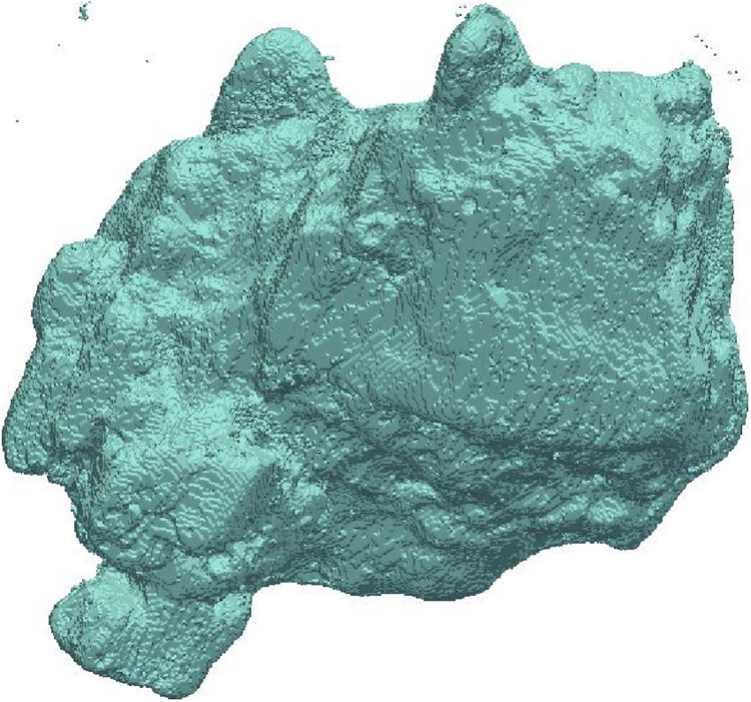
Vug space after acid flood.

Core is scanned in the medical CT scanner and vug space is reconstructed using Fiji-ImageJ (Fig. 2). The vug visualizations are compared to validate the repeatability of the system.

The extremities are not captured well in the reconstruction, but this can be attributed to the limited resolution of the 3D–printed vug space.

## Additional information

The glass bead proxies have been utilized in study of particle straining in porous media studies in hydrology and petroleum engineering. The repeatable proxy is a controllable parameter with respect to the naturally occurring rock samples, which can vary significantly in pore geometry and distribution.
